# Prevalence and predictors of depression, anxiety, and stress symptoms among patients with type II diabetes attending primary healthcare centers in the western region of Saudi Arabia: a cross-sectional study

**DOI:** 10.1186/s13033-019-0307-6

**Published:** 2019-07-16

**Authors:** Alhussain Alzahrani, Abdulrahman Alghamdi, Turki Alqarni, Reem Alshareef, Abdullah Alzahrani

**Affiliations:** 1 College of Medicine, King Saud Bin Abdulaziz University for Health Sciences, Jeddah, Saudi Arabia; 2 King Abdullah International Medical Research Center, Jeddah, Saudi Arabia; 3 Department of Family Medicine, Ministry of the National Guard-Health Affairs, King Abdulaziz Medical City, Jeddah, Saudi Arabia

**Keywords:** Diabetes, Diabetes mellitus type 2, Depression, Anxiety, Mental health

## Abstract

**Background:**

Type 2 diabetes mellitus (T2DM) is a prevalent health problem, not only globally, but also in Saudi Arabia. A growing body of literature suggests a bi-directional association between T2DM and various mental health disorders. This study aimed to investigate the prevalence and predictors of depression, anxiety, and stress among T2DM patients in the western region of Saudi Arabia.

**Methods:**

Between May and August 2018, a cross-sectional study was conducted among adult patients with T2DM in five public primary care centers in the western region of Saudi Arabia. Sociodemographic characteristics and symptoms of depression, anxiety, and stress were measured using the self-administered, previously validated Depression, Anxiety, and Stress Scale (DASS-21) questionnaire. Simple descriptive statistics were used. Forward binary logistic regression was used to identify predictors of depression, anxiety, and stress.

**Results:**

A total of 450 adults with T2DM were included (56.9% men; 43.1% women). The prevalence of depression, anxiety, and stress was 33.8%, 38.3%, and 25.5%, respectively. Major predictors of psychological distress were age, sex, the presence of comorbidities, duration since T2DM diagnosis, and serum level of hemoglobin A1c. Compliance with diabetes management measures and older age were the only protective factors.

**Conclusion:**

Patients with T2DM had significantly high rates of depression, anxiety, and stress. We recommend periodic screening of patients with T2DM for psychological distress using easy and inexpensive validated screening tools like the DASS-21 questionnaire. Further larger-scale studies are needed to investigate the causes and outcomes of these higher rates of psychological distress among Saudi patients with diabetes.

## Background

Diabetes mellitus is a chronic metabolic disease caused by impairment or deficiency in the production of insulin in the pancreas [1]. It is divided into type 1 and type 2 diabetes mellitus (T2DM). T2DM is clinically diagnosed when the patient presents with a fasting plasma glucose level of ≥ 7.0 mmol/L, a plasma glucose values ≥ 11.1 in a 2-h plasma oral glucose tolerance test of 75 g, or glycated hemoglobin A1c level (HbA1c) ≥ 6.5% [1]. It is well-known to be associated with multiple ophthalmological, renal, cardiovascular, neurological, and musculoskeletal complications. According to the World Health Organization (WHO), 422 million adults had diabetes in 2014, which accounted for 8.5% of the global population [2]. In Saudi Arabia, the prevalence of diabetes mellitus, according to the WHO, is estimated to be 14.4% among adults [3].

Depression is a serious and common illness which negatively affects how a person feels, thinks, and act [4]. People with depression might also present with anxiety, a feeling of tension and worried thoughts combined with physical changes [5], and stress, a relationship between a person and his or her environment that is described to be exceeding his or her resources thereby endangering his or her wellbeing [6]. These negative emotions affect the quality of life in all aspects, including sleep patterns, diet, education, career, relationship, and health, and also affect friends, family, and colleagues [7]. In 2015, the WHO estimated the prevalence of depression and anxiety disorders to be around 4.4% and 3.6%, respectively [8]. In Saudi Arabia, the prevalence of depression and anxiety disorders in 2017 was estimated to be around 4.5% and 4.3%, respectively [8].

Previous studies have investigated the association and prevalence of depression, anxiety, and stress among patients with T2DM in different countries [9–16]. A study observing 245,404 patients found that depression plays an important role in predicting the prognosis of chronic diseases [17]. It indicated that when depression was combined with a chronic disease, it was the greatest factor contributing to health deterioration compared to depression alone, chronic disease alone, and a combination of chronic diseases without depression [17]. In Saudi Arabia, few studies with small sample sizes have investigated the prevalence, characterization, and predictors of depression and anxiety only among T2DM patients. Previous studies have assessed the prevalence of depression with or without anxiety [18–27]. The prevalence of depression among T2DM patients ranged from 14.5 to 77.8%. Anxiety was reported in only two studies and was found to occur at a rate of 43.6% and 28% [25, 26].

Therefore, the aim of this study was to identify the prevalence and predictors of depression, anxiety, and stress among T2DM patients attending five primary health care centers in the western region of Saudi Arabia.

## Methods

This cross-sectional study was conducted between June and November 2018 in five primary healthcare centers of the Ministry of National Guards Health Affairs (MNGHA) in the western region of Saudi Arabia. The medical services of the MNGHA is composed of primary healthcare services scattered over the Saudi Arabia along with hospital and medical cities provide more advanced for all beneficiaries. The basic population consisted of all Saudi adults (≥ 18 and < 70 years old), men and women, who were diagnosed with T2DM more than 1 year ago. Those with a previous history of psychiatric illnesses or cognitive impairment were excluded, Ethical approval to conduct the study was obtained from King Abdullah International Medical Research Center (KAIMRC) (reference number: RYD-18-417780-108442). Informed consents were obtained from all participants.

The yearly average number of patients with diabetes who attend these primary healthcare centers of the MNGHA is around 15,000. This number was used to calculate the sample size needed for this study. Considering a 95% confidence interval (CI), a 5% margin of error, and a presumed prevalence of depression of 50% (based on data from the Alqassem region) [26], the minimum required sample size was calculated to be 375. Considering a 10% non-response rate, the final sample size was 450. We followed a quota sampling technique where 90 patients were selected from each center.

Data were collected by distributing a self-administered questionnaire composed of two parts. The first section addressed the sociodemographic data of each patient and the current status of diabetes mellitus indicated by findings such as the duration of diabetes, regularity of follow-up (every 3 to 6 months based on patient’s condition), most recent HbA1c, current regimen for diabetes management, and complications of diabetes. The second part screened for depression, anxiety, and stress using the previously-validated Arabic version of the Depression, Anxiety, and Stress Scale (DASS-21) questionnaire [28]. It consists of 21 items distributed on three scales for depression, anxiety, and stress, i.e. seven items each. Subjects are asked if they experience a set of symptoms for each scale during the last week. These items are designed to assess the symptoms of depression, anxiety, and stress on a scale from ‘0’ (does not apply to me) to ‛3’ (applies to me most of the time). Scores for each scale of the DASS-21 are calculated by summing the scores of the items and multiplying by 2 to match the original 42-question version of the questionnaire [28]. For depression, a score < 9 is ‘normal,’ 10–13 is ‘mild,’ 14–20 is ‘moderate,’ 21–27 is ‘severe,’ and > 27 is ‘extremely severe.’ For anxiety, a score < 7 is ‘normal,’ 8–9 is ‘mild,’ 10–14 is ‘moderate,’ 15–19 is ‘severe,’ and > 19 is ‘extremely severe.’ Finally, for stress, a score < 14 is ‘normal,’ 15–18 is ‘mild,’ 19–25 is ‘moderate,’ 26–33 is ‘severe,’ and > 33 is ‘extremely severe.’

Data management and analyses were performed with the Statistical Package for Social Sciences (SPSS, version 23.0.0.0, IBM, USA). Descriptive statistical analyses were performed for the study sample. Values were reported as proportions and percentages for categorical variables and as means and standard deviations or modes with ranges for continuous variables. Using participants’ characteristics as independent variables, a forward binary logistic regression was performed to assess the probability of being affected by any of the three forms of psychological distress (depression, anxiety, and stress). The results were reported as odds ratios (ORs) with 95% CIs. Statistical significance was considered at a p-value < 0.05.

## Results

### Baseline characteristics

A total of 450 patients agreed to participate and completed the survey. Of them, 256 (56.9%) were men, and 194 (43.1%) were women. The majority of participants were married (389; 86.4%) and had received some sort of education (ranging from primary school to advanced postgraduate degrees) (377; 83.8%). Approximately 170 (37.8%) participants were unemployed, and 191 (42.4%) had a monthly income less than < 5000 SR. A family history of depression, anxiety, or stress was present in only 32 (7.1%) respondents. Further details of the sociodemographic characteristics are provided in Table 1.

**Table 1:** Sociodemographic characteristics of the participants

Variable	Mean (± SD)/n (%)
Age (years)	56.9 (± 11.1)
Body mass index (kg/m^2^)	
< 18.5	3 (0.7)
18.5–24.9	77 (17.1)
25–29.9	144 (32)
30–34.9	129 (28.7)
35–39.9	58 (12.9)
> 40	39 (8.7)
Marital status	
Never married	3 (0.7)
Married	389 (86.4)
Widow/widower	41 (9.1)
Divorced/separated	17 (3.8)
Offspring	
No	15 (3.3)
Yes	435 (96.7)
Level of education	
No formal education	73 (16.2)
Primary school	72 (16)
Intermediate school	79 (17.6)
Secondary school	120 (26.7)
College	94 (20.9)
Higher education (Master’s, Ph.D.)	12 (2.7)
Occupation	
Retired	158 (35.1)
Unemployed	170 (37.8)
Employed	122 (27.1)
Income (in SR)	
< 5000	191 (42.4)
5000–10,000	138 (30.7)
10,001–15,000	85 (18.9)
15,001–20,000	20 (4.4)
> 20,000	16 (3.6)
Smoking status	
Smoker	195 (43.3)
Non-smoker	255 (56.7)
Comorbidities	
No	143 (31.8)
Yes	307 (68.2)
History of cancer	
No	437 (97.1)
Yes	13 (2.9)
Family history of depression, anxiety, or stress	
No	418 (92.9)
Yes	32 (7.1)

*SD* standard deviation, *SR* Saudi riyal

### Characteristics and status of T2DM

The median duration of T2DM among the participants was 8 (75) years. As shown in Table 2, approximately two-thirds of patients reported regular follow-up for their T2DM with their primary care physicians (288; 64%) and developed no T2DM-related complications (286; 63.8%). The most common T2DM management applied to respondents were lifestyle modifications accompanied by oral medications (252; 56%); the rate of compliance with these measures was reported to be 58.9%.

**Table 2:** Diabetes status of the participants

Variables	Median (range)/mean (± SD)/n (%)
Duration of diabetes (years)	8 (75)
Regularity of follow up	
Irregular	162 (36)
Regular	288 (64)
Most recent HbA1c level (%)	8 (1)
Complications of diabetes	
None	286 (63.8)
Diabetic retinopathy	35 (7.8)
Diabetic nephropathy	23 (5.1)
Cardiovascular complications	35 (7.8)
Diabetic foot ulcers/amputation	1 (0.2)
> 1 complications	69 (15.3)
Current diabetes management	
Lifestyle modifications	3 (0.7)
Lifestyle modifications + oral medications	252 (56)
Lifestyle modifications + oral medications + insulin	152 (33.8)
Lifestyle modifications + insulin	43 (9.6)
Compliance with diabetes management measures	
No	185 (41.1)
Yes	265 (58.9)

*SD* standard deviation, *HbA1c* glycated hemoglobin A1c

### Prevalence of depression, anxiety, and stress

Anxiety was the most common form of psychological distress present among the participants (171; 38%). Moreover, around 153 (33.8%) participants had depression, and 125 (25.3%) experienced stress. There was a female predominance among respondents regarding the three forms of mental health problems; women accounted for 57.9% of patients with depression, 54.4% of patients with anxiety, and 57% of patients with stress. The prevalence of the three forms of psychological distress based on severity is summarized in Table 3.

**Table 3:** Severity of diabetes, anxiety, and stress symptoms among the participants

Mental disorder	n	%
Depression		
Normal	298	66.2
Mild	42	9.3
Moderate	63	14.0
Severe	32	7.1
Extremely severe	15	3.3
Anxiety^a^		
Normal	276	61.7
Mild	60	13.4
Moderate	58	13.0
Severe	27	6.0
Extremely severe	26	5.8
Stress^a^		
Normal	333	74.5
Mild	38	8.5
Moderate	44	9.8
Severe	22	4.9
Extremely severe	10	2.2

^a^Missing data = 3

### Predictors of depression, anxiety, and stress

Table 4 shows the forward binary logistic regression models, including the significant predictors of depression, anxiety, and stress. Across all three models, a 1-unit increase in the most recent hemoglobin A1c level was associated with a 2.03-fold increase in the odds of having depression (95% CI 1.65–2.51), a 1.54-fold increase in having anxiety (95% CI 1.29–1.84), and a 1.55-fold increase in having stress (95% CI 1.29–1.85). In addition, the presence of comorbidity was associated with a 1.94-fold increase in the odds of having depression (95% CI 1.08–3.46) and a 1.65-fold increase in having anxiety (95% CI 1.01–2.69).

**Table 4:** Significant predictors of depression, anxiety, and stress on forward binary logistic regression

Independent variables	p	OR	95% CI	
			Lower	Upper
Depression				
Females (reference: males)	0.000	2.690	1.620	4.465
Age	0.000	0.950	0.925	0.977
Comorbidities	0.026	1.936	1.082	3.464
Duration of diabetes (years)	0.004	1.065	1.020	1.112
Most recent HbA1c level	0.000	2.032	1.645	2.511
Compliance with diabetes management measures	0.006	0.477	0.280	0.810
Anxiety				
Females (reference: males)	0.009	1.794	1.157	2.782
Comorbidities	0.043	1.654	1.017	2.689
Family history of chronic diseases	0.006	1.841	1.192	2.842
Most recent HbA1c level	0.000	1.542	1.290	1.842
Compliance with diabetes management measures	0.048	0.612	0.377	0.996
Stress				
Females (reference: males)	0.012	1.857	1.147	3.005
Family history of chronic diseases	0.000	2.385	1.473	3.860
Most recent HbA1c level	0.000	1.547	1.291	1.853
Compliance with diabetes management measures	0.020	0.527	0.308	0.903

*OR* odds ratio, *CI* confidence interval, HbA1c, glycated hemoglobin

Moreover, self-reported compliance with diabetes management measures was associated with a decreased odds of having depression (OR = 0.48, 95% CI 0.28–0.81), anxiety (OR = 0.61, 95% CI 0.38–0.99), or stress (OR = 0.53, 95% CI 0.31–0.90). Furthermore, female participants were 2.69 times more likely to have depression (95% CI 1.62–4.47), 1.79 times more likely to have anxiety (95% CI 1.16–2.78), and 1.86 times more likely to have stress (95% CI 1.45–3).

For depression alone, an increase in age was associated with a decreased odds of suffering from depression (OR = 0.95, 95% CI 0.93–0.98). Regarding the time since a T2DM diagnosis, a 1-unit increase was associated with a 1.07-fold increase in the probability of having depression (95% CI 1.02–1.11).

In the anxiety and stress models, those with a family history of chronic diseases were 1.84 times more likely to suffer from anxiety (95% CI 1.19–2.84) and 2.39 times more likely to suffer from stress (95% CI 1.47–3.86).

## Discussion

This study assessed the prevalence and predictors of depressions, anxiety, and stress among patients with T2DM in primary care settings in Saudi Arabia. Of the 450 participants, 38% exhibited anxiety, 33.8% showed depressive symptoms, and 25.3% suffered from stress. The main predictors of psychological distress were elevated hemoglobin A1c levels, presence of comorbidities, self-reported compliance with diabetes management, being a woman, family history of chronic diseases, duration since T2DM diagnosis. Advancing age and compliance on treatment were associated decreased odds of depression, anxiety and stress.

Similar to two studies of Saudi patients with diabetes that examined anxiety in comparison to other mental health problems, anxiety was the most common form of psychological distress [25, 26]. The prevalence of anxiety in our sample is similar to that found among T2DM patients in the central region of Saudi Arabia (43.6%) but higher than the prevalence reported among patients in the northern parts of Saudi Arabia (28.5%) [25, 26]. Another study conducted in Qatar using the DASS-21, revealed that more than half of the T2DM patients suffered from significant symptoms of anxiety [29].

The prevalence of depression among patients with T2DM in our study concurs with the rates of 34–37.9% found by several local studies [18–27]. In one study that was conducted among patients in secondary and tertiary hospitals, the prevalence of depression was substantially higher at around 78% using the Patient Health Questionnaire (PHQ-9) [24]. A lower prevalence of depression (22.4% and 20.68%) was observed in two other studies [22, 25]. This variation in the prevalence of depression might be attributed to different settings (i.e. primary versus secondary and tertiary healthcare centers) and sociodemographic characteristics of the participants. Despite these varying estimates, it is well documented that rates of psychological distress (e.g., anxiety and depression) are higher in patients with T2DM than in the general population [30].

In line with other studies, we also found that female sex was a predictive factor for depression, anxiety, and stress. Multiple factors, though we did not examine them, from different studies have been linked to this female predominance such as lack of social support as well as experiencing adverse life events [31, 32]. Additionally, our study revealed that an increased duration of diabetes was associated with an increased odds of having depression. Similar findings have been reported by several studies [9, 29]. Cumulative vulnerability regarding the time to develop diabetes-related complications is proposed as a possible mechanism underlying the association between the duration of diabetes and anxiety and depression.

The presence of comorbidities was also a strong predictor for anxiety and stress. The association between physical illnesses and anxiety has been proven in multiple studies concerning a wide range of physical complaints [33]. Moreover, a study conducted in 2017 on a similar population revealed a high prevalence of mental health disorders (57.3%) among patients with diabetes and hypertension attending primary health care centers [34]. Another study among T2DM patients showed that comorbidities were strong predictors of stress [35]. In addition, our findings corroborate those of previous studies where individuals with a family history of chronic diseases (e.g., T2DM and hypertension) were at risk of anxiety and stress [35, 36].

Our findings are also consistent with previous longitudinal and cross-sectional studies where poor glycemic control, expressed as elevated hemoglobin A1c, was associated with depression, anxiety, and stress [37, 38]. One possible explanation for this association was the finding of a meta-analysis of observational studies that depression was associated with higher rates of non-adherence to management plans for diabetes [39]. Anxiety and stress have also been associated with poor treatment compliance and implicated as predictors of poor glycemic control in multiple large-scale studies [38, 40].

In addition to compliance with diabetes management measures, advancing age was a protective factor against depression in our sample. Multiple longitudinal and community-based studies have similarly demonstrated that younger T2DM patients were at higher risk of depression [41, 42].

The strengths of this study lie in the larger sample size in comparison to previous studies. It is also the first, to our knowledge, to examine stress and the co-occurrence of depression, anxiety and stress among patients of diabetes in Saudi Arabia. However, this study has some potential limitations. First, the DASS-21 is only a screening tool for depression, anxiety, and stress symptoms. Second, the cross-sectional design is inadequate to assess the direction of the relationship between depression, anxiety, and stress and T2DM. In addition, the inclusion of controls in future studies can help to further elucidate the nature of the relationship between mental health disorders and T2DM.

## Conclusion

The findings of this study revealed high rates of depression, anxiety, and stress symptoms among patients with T2DM. Older age and compliance with diabetes management measures were significant protective factors. In comparison, female sex, elevated hemoglobin A1c, the presence of comorbidities, and a positive family history of chronic diseases were significant predictors of depression, anxiety, and/or stress. Periodic screening of patients with diabetes in primary healthcare settings for early signs of psychological distress using easy and inexpensive validated screening tools like the DASS-21 questionnaire is recommended. Further studies with larger sample sizes and control subjects need to be conducted to investigate the causes and outcomes of these higher rates of psychological distress among Saudi patients with diabetes.

## Data Availability

The datasets used and/or analysed during the current study are available from the corresponding author on reasonable request.

## Abbreviations

HbA1c: hemoglobin A1c
CI: confidence interval
DASS-21: Depression, Anxiety, and Stress Scale
MNGHA: Ministry of National Guards Health Affairs
OR: odds ratio
T2DM: type 2 diabetes mellitus
WHO: World Health Organization
